# Development of a European job exposure matrix (EuroJEM) for psychosocial exposures and their association with diagnosed depression in register-based cohorts

**DOI:** 10.5271/sjweh.4279

**Published:** 2026-05-01

**Authors:** Laura Salonen, Daniel Falkstedt, Kuan-Yu Pan, Maria Albin, Ingrid Sivesind Mehlum, Karina Undem, Taina Leinonen, Svetlana Solovieva

**Affiliations:** 1 Finnish Institute of Occupational Health, Helsinki, Finland.; 2 Unit of Occupational Medicine, Institute of Environmental Medicine, Karolinska Institutet, Stockholm, Sweden.; 3 Division of Occupational and Environmental Medicine at Lund University, Lund, Sweden.; 4 National Institute of Occupational Health, Oslo, Norway.; 5 Bispebjerg, Frederiksberg Hospitals, Copenhagen, Denmark; University of Copenhagen, Copenhagen, Denmark.; 6 University of Oslo, Oslo, Norway.

**Keywords:** decision authority, epidemiology, harmonization, job demand, job strain, meta-analysis

## Abstract

**Objectives:**

This study developed a European job exposure matrix (EuroJEM) for psychosocial work factors in order to examine their prospective associations with diagnosed depression in three register-based Nordic cohorts.

**Methods:**

National, gender-specific psychosocial JEM from Finland, Norway, and Sweden were evaluated for similarities in exposures, exposure definitions, and occupational coding. The EuroJEM harmonized two exposures: quantitative job demands and decision authority. Disagreements on exposure categories across the national JEM were addressed among experts. Associations between exposures and diagnosed depression were examined across three register-based cohorts.

**Results:**

The EuroJEM provides gender-specific exposure categories, based on the proportion of workers exposed, for 371 ISCO-88 (COM – European version of the International Standard Classification of Occupations) occupational codes. All associations were similar across the three cohorts, except for medium-high / high likelihood of exposure to high job demands among women. The pooled hazard ratios (pHR) for depression among workers with a medium-high / high likelihood of exposure to low decision authority had pHR of 1.50 [95% confidence interval (CI) 1.33–1.68] among men and 1.28 (95% CI 1.22–1.35) among women. High strain jobs had pHR of 1.19 (95% CI 1.15–1.24) and 1.07 (95% CI 1.01–1.14) and active jobs 0.79 (95% CI 0.72–0.87) and 0.86 (95% CI 0.79–0.94) among men and women, respectively. The associations between job demands and depression were less clear, especially among women.

**Conclusion:**

We found consistent associations between diagnosed depression and EuroJEM-based psychosocial exposures. Especially decision authority and job strain indicate a good performance of this JEM. The performance for job demands may be suboptimal.

Psychosocial work exposures are linked to numerous health outcomes (1, 2). According to a recent umbrella review (3), several adverse psychosocial exposures increase the risk of mental disorders, although the magnitude of the risk was generally low. Limited evidence for a causal association between psychosocial exposures and depressive episodes was found in another systematic review (4). The evidence for causal association is considered limited because in most studies both psychosocial exposures and depression are based on self-reports, a situation that is referred to as common method variance bias (5, 6). To improve the certainty of the evidence, two reviews (1, 2) have recommended future studies apply independent assessment of exposures and outcome(s) and depend less on self-reported exposures; they also called for more studies with aggregated self-reported exposures either at the job group or organizational level.

Job exposure matrices (JEM) provide standardized exposure estimates that are not biased by outcome status or recall mistakes by linking exposure estimates to the job titles. JEM are particularly useful in large scale epidemiological studies, where individual-level exposure data are not available (7–9). However, they neglect both between- and within-worker variation in a job due to over time changes, as well as variation in tasks, activities and work processes/organization (10) and therefore may result in misclassified exposure assignments for a considerable proportion of workers.

Several JEM for psychosocial exposures have been developed (11–22). The existing national psychosocial JEM vary widely in exposures and definitions, with dimensions of Karasek’s model (13) being the most common. For example, some JEM for job demands include emotional demands (11, 14) while others do not (15–17). Similarly, some JEM include skill discretion in defining decision authority (17–21) and others do not (14, 15).

National JEM, however, have reduced generalizability and applicability. JEM built in one country are rarely directly applicable in other countries, and constructing a national JEM is demanding and resource consuming. A successful harmonization of national JEM can yield standardized exposure measures across regions and time periods (23). The main added value of such a harmonized JEM is that it could be used in large-scale studies where (access to) a national JEM is lacking. However, harmonization is challenging due to variations in occupational coding and definitions of exposures. To address this need, the Exposome Project for Health and Occupational Research (www.ephor-project.eu) aims to develop European JEM (EuroJEM) by harmonizing exposure definitions, occupational coding and exposure estimates of national JEM and increase their applicability in large occupational cohorts (24, 25).

In this study, we aimed to (i) develop a gender-specific European JEM for psychosocial exposures to be used in large epidemiological studies that lack individual exposure information and (ii) examine prospective associations of JEM-based exposures with diagnosed depression in Finland, Norway and Sweden.

## Methods

### Selection of existing JEM for psychosocial exposures

The existing psychosocial JEM in Europe were identified via search of published documents in PubMed as well as Google Scholar and complemented by JEM registered in the inventory of existing JEM of the OMEGA-NET COST Action (occupationalexposuretools.net). In total, 12 JEM for psychosocial exposures from seven countries, of which 6 were sex-specific, were found (supplementary material, www.sjweh.fi/article/4279, table S1). All identified JEM were constructed based on self-reported exposures and had a mean score value assigned for each occupational code. Most of the JEM included quantitative job demands and decision authority. The other exposures covered by JEM were emotional job demands, monotonous work, psychosocial resources and social support. The majority of the JEM used national occupational classification systems based on the ISCO-88 COM, ie, the European version of the International Standard Classification of Occupations (COMmision) 1988 (ISCO-88) for use within the European Union. JEM with similar exposures and their definitions as well as occupational coding systems were selected for harmonization (see description of these JEM in supplementary methods S1). Two exposures, decision authority and quantitative job demands, were selected for the development of EuroJEM for psychosocial factors. These exposures were comparable across three national gender-specific JEM (supplementary table S2).

### Development of the EuroJEM for psychosocial exposures

The exposure measures of the national JEM were constructed as summary scores for decision authority and job demands by occupation. In the Swedish JEM, the scores reflected the average level of exposure within each occupation, whereas in the Finnish and Norwegian JEM, they were based on the proportion of workers exposed. Prior to the development of EuroJEM, the national JEM were revised so that all expressed exposure as the proportion of exposed workers within the occupation. The individual responses for each item measuring decision authority and job demands (described in supplementary table S2) were summed. Individuals were considered ‘exposed’ if their sum score was above median. Then, within each occupational code, for women and men separately, the proportion of workers exposed to low decision authority and high job demands in each national cohort was calculated.

To develop the psychosocial EuroJEM, we harmonized the occupational codes into the same coding system [ISCO-88 (COM)]. For this, the occupational codes of the national JEM from the three Nordic countries were transcoded to ISCO-88 (COM) using a recently developed Nordic crosswalk, allowing for comparability of occupational titles across Nordic countries (26).

Both low decision authority and high job demands were harmonized within each occupation as follows. First, in each national JEM we categorized the proportion of exposed workers into four categories: low (0–24%); medium (25–49%); medium-high (50–74%) and high (50–100%) likelihood of exposure. The distribution of exposure categories in the national JEM is shown in supplementary table S3. In epidemiological research, the term “likelihood of exposure” is often used interchangeably with the “proportion of exposed individuals” within a defined group or population. It does not refer to causal connotation but rather indicates the individual’s risk of exposure within an occupation.

Existing psychosocial JEM usually use occupation-specific summary or mean exposure scores of psychosocial exposures (eg, job demands, job control) which are often categorized (using a median as cut-off, or tertiles, or quartiles) to distinguish between high and low measures in the analyses. The exposure scores do not account for the variability around the mean, the differences in the variability between occupations, or the size of the occupation. As a result, most occupations would be allocated around the mean value but the distribution of scores would vary. Mean score may give a false impression that all workers within a specific occupation would have “an average exposure score X”, when in reality only some are exposed while others are never exposed. The score distributions are always based on the study sample used for development of the JEM and, for the same occupation score, measures may vary considerably across different psychosocial JEM due to differences in questionaries and study populations used. All the above-mentioned reasons reduce comparability between the JEM and make their harmonization difficult. The metrics of the three national JEM used for construction of the EuroJEM were converted from occupation-specific score to proportion of exposed within the occupation prior to harmonization.

Then, we examined similarities and differences in the exposure category for each occupation and gender across the national JEM (supplementary table S4). If the exposure category for an occupation was the same in all JEM, this exposure category was assigned to the occupation in the EuroJEM. To be considered as having a “high likelihood of exposure”, the occupational groups had to be in the high exposure category in at least two national JEM. Disagreements between the national JEM were resolved by a consensus procedure of a researcher panel consisting of at least one researcher from each country with expertise in occupational medicine and work environment. The procedure is explained in detail in the supplementary methods S2.

Concordance between the national JEM and the EuroJEM was checked by comparing distribution of exposure categories between each national JEM and the EuroJEM (supplementary table S5). The EuroJEM thus provides four exposure categories based on the proportion of workers exposed to low decision authority and high job demands.

We further used the EuroJEM to construct job strain measures (low decision authority and low job demands = passive; low decision authority and high job demands = high strain; low job demands and high decision authority = low strain; high job demands and high decision authority = active).

### Register-based cohorts

We used individual-level data from three national register-based cohorts: the State of the Labor Force in Finland (SLFF) (27), the Norwegian Working-Age Cohort (Nor-Work) (28) and the Swedish Work, Illness, and Labor Market Participation Cohort (SWIP) (29). The description of the cohorts is found in supplementary methods S3.

For all cohorts, we included individuals who were employed wage-earners according to their main economic activity and socioeconomic status and aged 25–55 at baseline (ie, on the last day of 2005, 2007, 2010 for Sweden, Norway and Finland, respectively). Those who did not have information on the occupational title at baseline or within two years prior to baseline were excluded. We also excluded individuals in the ‘armed forces’ occupational group, as this group was not included in the EuroJEM. The study populations for Finland, Norway and Sweden consisted of 2 921 269 (50.6% women), 1 657 139 (48.24% women), and 1 371 871 (51.2% women) individuals, respectively, in total, 5 950 279 individuals.

We used ISCED (isced.uis.unesco.org/) to categorize education into three categories following the ISCED levels for each country: primary (categories 0–2), secondary (categories 3–5) and tertiary (categories 5–7).

### Diagnosed depression

The outcome was based on the first visit to specialized health care due to depression (International Classification of Diseases version 10 (ICD-10) codes F32 or F33, either primary or secondary diagnosis) using in- and out-patient (specialized health care) registers during 2006–2020, 2008–2021 and 2011–2021 for Swedish, Norwegian and Finnish cohorts, respectively. We did not control for previous healthcare visits.

### Statistical analyses

We assigned the gender- and occupation-specific exposure values from the EuroJEM to the participants of each national cohort and examined the associations of the EuroJEM for psychosocial exposure measures with diagnosed depression using Cox regression. In the analysis, medium-high and high likelihood of exposure were merged due to a small number of observations in the latter group, resulting in three exposure categories. The reference group for low decision authority and high job demands was the 'low likelihood of exposure' group, while for job strain, it was the 'low strain' group. The hazard ratios (HR) and their 95% confidence intervals (CI) were adjusted for age only (model 1) and in combination with education (model 2). All persons were followed up until the first healthcare visit due to depression, emigration, death or end of follow-up, whichever came first. All analyses were performed within each national cohort separately for men and women.

We pooled cohort-specific risk estimates for the association of high job demands, low decision authority and job strain with diagnosed depression from model 2 in random-effects meta-analysis and assessed heterogeneity with the I^2^ and τ statistic and Cochrane’s Q test (30).

For sensitivity analyses we repeated analyses using the original four groups of exposure categories to explore potential dose-response patterns. In addition, we conducted meta-analyses for model 1 to examine the impact of adjustment on the pooled HR.

## Results

### Description of the EuroJEM

Gender-specific psychosocial work exposures for 371 4-digit ISCO-88 (COM) codes were included in the EuroJEM. The distribution of exposure categories across occupational groups in the EuroJEM was similar for men and women (table 1). In both genders, most of the occupations were classified as having a medium likelihood of exposure (25–49%) to high job demands and/or low decision authority. Only a few occupations belong to the category of high likelihood of exposure (75–100%) (table 1 and supplementary table S6).

**Table 1:** Number of ISCO-88 (COM) codes by exposure categories in EuroJEM among men and women.

Exposure/categories		Men		Women
		N (%)		N (%)
Total		374		374
High job demands				
	Low likelihood (0–24%)	41 (11.0)		47 (12.6)
	Medium likelihood (25–49%)	237 (63.4)		229 (61.2)
	Medium–high likelihood (50–74%)	91 (24.3)		95 (25.4)
	High likelihood (75–100%)	5 (1.3)		3 (0.8)
Low decision authority				
	Low likelihood (0–24%)	47 (12.6)		53 (14.2)
	Medium likelihood (25–49%)	168 (44.9)		172 (46.0)
	Medium-high likelihood (50–74%)	153 (40.9)		142 (40.0)
	High likelihood (75–100%)	6 (1.6)		7 (1.9)

### Description of the cohorts

The three national cohorts had some differences in the distribution of sociodemographic factors. The mean ages were 40.0 years among Finnish and Norwegian men and women, 39.9 years among Swedish men and 40.3 years among Swedish women. The Norwegian cohort had more primary-educated participants than Finland and Sweden, and Finland had the highest proportion of tertiary-educated individuals (table 2). Among men, the most common major occupations were 'craft and related workers' in the Finnish cohort and 'technicians and associate professionals' in both the Norwegian and Swedish cohorts. Among women, the service and sales workers were the most common major occupational group in all cohorts.

**Table 2:** Descriptive characteristics of the study populations and proportions of workers in different categories of psychosocial exposures based on EuroJEM.

		Men						Women				
		Finnish N=669 597		Norwegian N=764 400		Swedish N=1 337 271		Finnish N=702 274		Norwegian N=728 838		Swedish N=1 446 794
		%		%		%		%		%		%
Age groups												
	25–29	15.9		14.1		14.5		14.3		14.0		13.9
	30–39	31.9		34.6		35.0		29.4		34.0		33.6
	40–49	33.1		33.6		32.6		34.9		33.9		33.4
	50–55 years	19.1		17.6		17.9		21.5		18.2		19.1
Education												
	Primary	12.0		17.5		13.1		6.8		16.3		8.7
	Secondary	50.4		44.3		51.7		39.7		37.2		47.9
	Tertiary	37.6		38.2		35.2		53.5		46.5		43.4
Major occupational groups												
	Legislators, senior officials, and managers	5.2		10.2		6.6		2.3		5.3		3.0
	Professionals	19.3		16.4		17.7		21.3		20.2		20.0
	Technicians and associate professionals	18.4		17.7		18.5		24.7		23.6		20.7
	Clerks	4.2		6.3		5.9		12.0		10.3		12.7
	Service and sales workers	8.2		10.9		9.9		25.7		30.5		32.2
	Skilled agricultural and fishery workers	1.2		1.0		1.1		0.9		0.3		0.4
	Craft and related trades workers	20.3		16.6		16.8		1.7		1.0		0.9
	Plant and machine operators and assemblers	15.8		13.9		17.5		3.7		2.7		3.2
	Elementary occupations	7.3		6.9		6.0		7.8		6.2		6.9
High job demands (% likelihood)												
	Low (0–24%)	4.4		5.3		7.1		3.7		3.3		7.9
	Medium (25–49%)	68.6		68.5		70.2		65.0		66.7		69.1
	Medium–high / high (0–100%)	27.5		26.2		22.9		32.5		30.0		23.8
Low decision authority (% likelihood)												
	Low (0–24%)	8.1		12.6		9.1		5.8		7.2		9.9
	Medium (25–49%)	54.8		54.5		54.0		46.7		46.8		53.2
	Medium–high / high (50–100%)	37.1		32.9		36.9		47.6		46.0		36.9

Around 3–8% of the workers were characterized as having a low likelihood of high job demands, with the highest proportion found in the Swedish cohort. Around 23–33% had a medium-high / high likelihood of high job demands, the highest proportion being in the Finnish cohort. Between 6–13% had a low likelihood of low decision authority, with the highest proportion found in the Norwegian and Swedish cohorts, and 33–48% had a medium-high / high likelihood of exposure to low decision authority, the highest proportion being in the Finnish cohort. The maximum follow-up times were 11, 14 and 15 years in the Finnish, Norwegian and Swedish cohorts, respectively.

In total, 4.9%, 7.2%, and 5.6% of the Finnish, Norwegian and Swedish cohorts, respectively, were diagnosed with depression during the follow-up (table 3). The prevalence of depression was higher among women than men and among younger than older workers.

**Table 3:** The prevalence and the 95% confidence intervals (CI) of diagnosed depression by country, gender and age group.

		Men						Women				
		Finland		Norway		Sweden		Finland		Norway		Sweden
		Prevalence (95% CI)		Prevalence (95% CI)		Prevalence (95% CI)		Prevalence (95% CI)		Prevalence (95% CI)		Prevalence (95% CI)
All		3.7 (3.6–3.8)		5.6 (5.5–5.6)		4.5 (4.5–4.5)		6.2 (6.1–6.3)		8.7 (8.7–8.8)		6.8 (6.8–6.8)
Age groups												
	25–29	4.2 (4.1–4.3)		7.0 (6.8–7.1)		5.1 (5.0–5.2)		8.0 (7.8–8.2)		11.9 (11.7–12.1)		8.6 (8.5–8.7)
	30–39	3.8 (3.7–3.9)		6.1 (6.0–6.2)		4.4 (4.4–4.5)		6.8 (6.7–6.9)		9.8 (9.7–10.0)		7.2 (7.1–7.3)
	40–49	3.7 (3.6–3.8)		5.2 (5.2–5.3)		4.5 (4.5–4.6)		5.8 (5.7–5.9)		8.0 (7.8–8.1)		6.5 (6.4–6.5)
	50–55	3.2 (3.1–3.3)		4.0 (3.9–4.1)		4.0 (3.9–4.1)		4.9 (4.8–5.0)		5.7 (5.6–5.8)		5.3 (5.3–5.4)

### Associations of the EuroJEM for psychosocial exposure measures with diagnosed depression

Table 4 shows the association between EuroJEM-based psychosocial exposures and diagnosed depression among men. In the age- and education-adjusted model 2, a medium compared to low likelihood of exposure to high job demands was associated with an increased risk of depression (HR 1.06–1.23). Contrarily, a medium-high / high likelihood of exposure to high job demands was associated with a decreased risk of depression (HR 0.89–0.98). The pooled estimated risks of the three cohorts were HR 1.11 (95% CI 1.03–1.20) and HR 0.89 (95% CI 0.79–1.00) for medium and medium-high / high likelihood of exposure, respectively.

**Table 4:** Associations between EuroJEM-based exposures and diagnosed depression by national cohort **among men**. Hazard ratios (HR) and 95% confidence intervals (CI).

Exposure		Model 1: Age-adjusted						Model 2: Age- and education-adjusted						Meta-analysis (Model 2)						
		Finnish		Norwegian		Swedish		Finnish		Norwegian		Swedish								
		HR (95% CI)		HR (95% CI)		HR (95% CI)		HR (95% CI)		HR (95% CI)		HR (95% CI)		HR (95% CI)		I^2%^		Q (P-value)		τ
High job demands (% likelihood)																				
	Low (0–24%)	1.00		1.00		1.00		1.00		1.00		1.00		1.00						
	Medium (25–49%)	1.16 (1.09–1.23)		1.01 (0.96–1.05)		1.01 (0.98–1.04)		1.23 (1.15–1.31)		1.06 (1.01–1.10)		1.07 (1.04–1.11)		1.11 (1.03–1.20)		81.3		16.1 (0.0003)		0.06
	Medium-high / high (50–100%)	0.80 (0.75–0.86)		0.82 (0.78–0.86)		0.69 (0.67–0.71)		0.98 (0.92–1.05)		0.91 (0.87–0.95)		0.80 (0.77–0.83)		0.89 (0.79–1.00)		91.5		35.5 (< 0.0001)		0.10
Low decision authority (% likelihood)																				
	Low (0–24%)	1.00		1.00		1.0		1.00		1.00		1.00		1.00						
	Medium (25–49%)	1.29 (1.22–1.36)		1.22 (1.17–1.26)		1.51 (1.46–1.57)		1.13 (1.07–1.20)		1.18 (1.14–1.22)		1.38 (1.33–1.44)		1.23 (1.09–1.38)		93.5		46.1 (< 0.0001)		0.10
	Medium-high / high (50–100%)	1.67 (1.60–1.79)		1.57 (1.52–1.63)		1.91 (1.84–1.98)		1.36 (1.28–1.44)		1.46 (1.41–1.51)		1.68 (1.62–1.75)		1.50 (1.33–1.68)		93.3		44.8 (< 0.0001)		0.10
Job strain																				
	Low	1.00		1.00		1.0		1.00		1.00		1.00		1.00						
	Passive	1.20 (1.17–1.24)		1.28 (1.25–1.30)		1.21 (1.19–1.23)		1.14 (1.11–1.17)		1.22 (1.19–1.24)		1.17 (1.15–1.19)		1.18 (1.14–1.22)		83.0		17.6 (0.0001)		0.03
	Active	0.71 (0.70–0.74)		0.82 (0.80–0.84)		0.68 (0.66–0.70)		0.81 (0.76–0.84)		0.84 (0.82–0.87)		0.73 (0.72–0.75)		0.79 (0.72–0.87)		95.3		63.3 (< 0.0001		0.09
	High	1.12 (1.07–1.17)		1.20 (1.15–1.25)		1.17 (1.09–1.25)		1.17 (1.12–1.22)		1.23 (1.18–1.28)		1.16 (1.08–1.24)		1.19 (1.15–1.24)		11.9		3.4 (0.8210)		0.02

Low decision authority was associated with an increased risk of depression, following a dose–response pattern. This risk varied for medium (HR 1.13–1.38) and for medium-high / high (HR 1.36–1.68) likelihood of exposure in the age- and education-adjusted models. The pooled estimated risks were HR 1.23 (95% CI 1.09–1.38) and HR 1.50 (95% CI 1.33–1.68) for medium and medium-high / high likelihood of exposure, respectively.

Compared to men in low strain jobs, men in passive (HR 1.14–1.22) or high strain (HR 1.16–1.23) jobs had a higher risk of depression, and those in active jobs (HR 0.73–0.84) had a lower risk of depression controlling for age and education. The pooled estimated risks were HR 1.18 (95% CI 1.14–1.22), HR 0.79 (95% CI 0.72–0.87) and HR 1.19 (95% CI 1.15–1.24) for passive, active and high strain jobs, respectively.

In general, the associations were slightly stronger in the age-adjusted model 1, ie, before having further adjusted for education in model 2.

As among men, a medium likelihood of exposure to high job demands increased the risk of depression among women in all cohorts in the age- and education-adjusted model 2 (table 5). However, differing from men, a medium-high / high likelihood of exposure was associated with a decreased risk only in the Swedish cohort (HR 0.93), and instead with an increased risk in the Norwegian (HR 1.15) and Finnish (HR 1.17) cohorts. The pooled estimated risk of the three cohorts was HR 1.17 (95% CI 1.05–1.30) and HR 1.08 (95% CI 0.92–1.26) for medium and medium-high / high likelihood of exposure, respectively.

**Table 5:** Associations between EuroJEM-based exposures and diagnosed depression by national cohort **among women**. Hazard ratios (HR) and 95% confidence intervals (CI).

Exposure		Model 1: Age-adjusted						Model 2: Age- and education-adjusted						Meta-analysis (Model 2)						
		Finnish		Norwegian		Swedish		Finnish		Norwegian		Swedish								
		HR (95% CI)		HR (95% CI)		HR (95% CI)		HR (95% CI)		HR (95% CI)		HR (95% CI)		HR (95% CI)		I^2%^		Q (P-value)		τ
High job demands (% likelihood)																				
	Low (0–24%)	1.00		1.00		1.00		1.00		1.00		1.00		1.00						
	Medium (25–49%)	1.22 (1.16–1.29)		1.19 (1.13–1.25)		1.00 (0.98–1.02)		1.25 (1.18–1.32)		1.21 (1.15–1.26)		1.07 (1.04–1.09)		1.17 (1.05–1.30)		92.5		42.1 (< 0.0001)		0.09
	Medium-high / high (50–100%)	0.96 (0.91–1.02)		1.10 (1.05–1.15)		0.75 (0.73–0.77)		1.17 (1.10–1.23)		1.15 (1.10–1.21)		0.93 (0.90–0.96)		1.08 (0.92–1.26)		96.2		78.9 (< 0.0001)		0.14
Low decision authority (% likelihood)																				
	Low (0–24%)	1.00		1.00		1.0		1.00		1.00		1.00		1.00						
	Medium (25–49%)	1.31 (1.25–1.38)		1.21 (1.17–1.25)		1.28 (1.25–1.31)		1.21 (1.15–1.27)		1.18 (1.14–1.22)		1.24 (1.21–1.27)		1.21 (1.17–1.25)		45.7		5.5 (0.0631)		0.02
	Medium-high / high (50–100%)	1.60 (1.52–1.68)		1.29 (1.24–1.33)		1.37 (1.34–1.41)		1.37 (1.31–1.44)		1.26 (1.22–1.30)		1.24 (1.21–1.27)		1.28 (1.22–1.35)		79.3		14.5 (0.0007)		0.04
Job strain																				
	Low	1.00		1.00		1.0		1.00		1.00		1.00		1.00						
	Passive	1.17 (1.15–1.20)		1.07 (1.05–1.09)		1.04 (1.02–1.05)		1.14 (1.11–1.17)		1.06 (1.04–1.08)		1.00 (0.98–1.01)		1.06 (0.99–1.14)		96.1		76.6 (< 0.0001)		0.06
	Active	0.81 (0.78–0.83)		0.90 (0.88–0.93)		0.73 (0.72–0.75)		0.81 (0.78–0.94)		0.93 (0.91–0.96)		0.84 (0.82–0.86)		0.86 (0.79–0.94)		91.1		33.9 (< 0.0001)		0.07
	High	1.05 (1.01–1.08)		1.03 (1.01–1.06)		0.89 (0.86–0.92)		1.16 (1.08–1.24)		1.07 (1.04–1.10)		1.01 (0.98–1.05)		1.07 (1.01–1.14)		79.1		14.4 (0.0008)		0.05

Similar to men, we found a dose–response pattern in the association between decision authority and depression among women. In the age- and education-adjusted models, a medium compared to low likelihood of exposure was associated with depression (HR 1.18–1.24), and the corresponding HR for medium-high / high likelihood were 1.24–1.37. The pooled estimated risks were HR 1.21 (95% CI 1.17–1.25) and HR 1.28 (95% CI 1.22–1.35) for medium and medium-high / high likelihood of exposure, respectively.

Passive and high strain jobs were associated with an increased risk of depression compared to low strain jobs only in the Norwegian and Swedish cohorts, with age-and education-adjusted HR of 1.06–1.14 for passive and 1.07–1.16 for high strain jobs. Active jobs were also among women associated with a decreased risk of depression in all cohorts, with HR of 0.81–0.93. The pooled estimated risks were 1.06 (95% CI 0.99–1.14), 0.86 (95% CI 0.79–0.94) and 1.07 (95% CI 1.01–1.14) for passive, active and high strain jobs, respectively.

Among women, comparing models 1 and 2 (ie, before and after adjusting for education), it was found that some of the associations with high job demands changed from negative to positive in the Finnish and Swedish cohorts. Associations related to passive and high strain jobs changed from significant to non-significant in the Swedish cohort.

The absolute amount of between-study variance (τ) showed that all estimated associations were similar across cohorts, except for medium-high / high likelihood of exposure to high job demands and depression among women. Heterogeneity of estimated associations of high job demands and job strain with depression across the cohorts is larger for women than men. Heterogeneity of estimated associations between low job control and depression across the cohorts is larger for men than women. Keeping in mind that the lower the value, the better the JEM performance, we can say that EuroJEM has the best performance for medium likelihood of exposure to low job control among women and high strain job among men.

### Sensitivity analyses

The sensitivity analyses using four exposure categories resulted in very similar findings. The main difference was found among women, where – in the four-category analysis – high likelihood of exposure to high job demands was associated with a decreased risk of depression (supplementary tables S7–S9). This was not captured when using three exposure categories. However, this exposure group consisted only of three occupations. We also examined the possible impact of adjustment for education, by comparing pooled estimates and τ-values between models 1 and 2. In both genders, the pooled HR for low decision authority decreased and for high job demands increased after further adjusting for education (supplementary tables S10–S11). However, the τ-values for associations in model 1 were larger than corresponding values in model 2.

## Discussion

Within the EPHOR project, we developed a gender-specific European psychosocial JEM (EuroJEM), measuring high quantitative demands and low decision authority, by harmonizing existing national JEM from Finland, Norway and Sweden. We utilized it to test prospective associations of the exposures with diagnosed depression in three national register-based cohorts.

Our findings revealed consistent associations of low decision authority and job strain with depression across all register-based cohorts. Specifically, workers with a medium likelihood of exposure to low decision authority faced an increased risk of depression, with the risk further elevated among those with a medium-high / high likelihood of exposure. Both men and women in high strain and passive jobs generally had an increased risk of depression compared to low strain jobs. Regarding job demands, the results varied by gender, country cohorts and models. In the age- and education-adjusted models, a medium likelihood of exposure to high job demands was associated with an increased risk of depression and medium-high / high likelihood of exposure decreased the risk of depression compared to low likelihood of exposure.

The EuroJEM differs from the existing psychosocial JEM both conceptually and methodologically. The main conceptual differences between national JEM and the EuroJEM are related to the exposure data sources and standardized international coding system. The EuroJEM was constructed via harmonization exposure definitions and occupational coding of national JEM, combining self-reported and expert judgement approaches. While national JEM rely on self-reported exposure information, EuroJEM’s exposure measures were assigned based on agreement of measures from the three JEM through researcher consensus. Methodologically the main difference between the EuroJEM and national JEM is related to the exposure metric, since the metric of the EuroJEM is semi-quantitative and the exposure categories are based on “proportion of exposed” within the occupational group, instead of mean scores (see detailed explanation in the Methods section).

We examined associations between exposures assessed by JEM and diagnosed depression, utilizing data from three large register-based cohorts, standardizing study design, register-based outcome and analytical approach across the cohorts. However, the difference between cohorts remained in the prevalence of depression and the occupational compositions, which induced differences in the distribution of exposure categories.

We observed larger similarities in the risk estimates between the three cohorts for low decision authority and job strain than for high job demands. Our findings align with previous results, indicating a better performance for job control and job strain than for job demands in detecting associations between these exposures and depression (3, 4, 6, 17, 20, 21), and tendency for better prediction of health outcomes (including depression) among men than women (14, 15, 17, 22, 31, 32).

Women had a higher prevalence of depression than men, and its association with psychosocial EuroJEM was stronger among men. Similar findings have been reported by previous studies, and they have provided some plausible explanations for this, such as differences in occupational segregation and the habits of reporting symptoms and illnesses between genders (15, 32, 33).

Since the exposure metrics of the EuroJEM differ from those in other psychosocial JEM, our results cannot be directly compared with those previously published. Furthermore, noticeable variation of effect estimates and inconsistency between the studies employing JEM due to heterogeneity of exposure measures and categorization used in the analyses make it difficult to compare results from different studies.

Nevertheless, aligning with our findings, previous studies reported an increased risk of depression among employees with low job control in at least one gender (9, 11, 15, 22, 34). In the current study, low decision authority was associated with increased risk of depression in both genders, following a dose–response pattern among men.

Our results on job strain showing an association between high strain job and increased risk of depression (compared to low strain) align with previous findings, based on studies using self-assessed exposures (32, 35–37) and JEM (15, 22, 32, 37). As an exception, among women such an association was only found in the Finnish and Norwegian cohorts, while the risk in the Swedish cohort was close to unity. Some previous studies also have failed to show such association in women (11, 22, 32, 38).

Regarding job demands, we found that Swedish men and women, and Norwegian men with medium-high / high likelihood of exposure to high job demands had a decreased risk of depression, while medium compared to low likelihood of exposure increased the risk of depression in all three cohorts and both genders. Previous JEM-based studies have found an increased risk of depression among women with medium demands (only among women) (11), but a decreased risk among those with medium-high and high demands (11) and in any category higher than the lowest category (15). The decreased risk of depression may reflect a healthy worker effect, where individuals with health problems are less likely to gain and/or maintain positions or employment in jobs with high job demands. It has been argued that jobs that are classified as having high demands also provide workers’ with challenges and opportunities that may decrease the risk of depression (15, 39). Previous studies on high job demands have reported mixed results. Studies based on self-reports have found null associations (40, 41) and that high job demands increase the risk of depression (41, 42). Especially job demands vary between individuals considerably more than between occupations (8, 13). Due to these reasons the EuroJEM, as well as other existing JEM, show limited performance for job demands.

The relatively small difference in risk estimates across the three cohorts might be explained by potential exposure misclassification due to reliance on occupational codes and crosswalks, outcome misclassification due to possible differences in disease classification codes, differences in the compositional structure of cohorts, and completeness of the register data. While pooled risk estimates based on few cohorts may not provide best evidence of the associations between exposures and depression, it provides useful information on the direction, size and consistency of associations. The pooled estimates suggest that the risk of depression is elevated among employees exposed to low decision authority and those in high strain jobs, while high job demands do not affect depression. Similar associations were found in published meta-analyses for high strain job [odds ratio (OR) 1.27, 95% CI 1.04–1.55 (36)], and high job demands [OR 1.08, 95% CI 0.98–1.19 (4)], but not for low decision authority [OR 0.93, 95% CI 0.77–1.13 (4)].

The EuroJEM has limitations common to all JEM (9), including its inability to capture individual exposure variation within occupations, temporal task variation, and systematic misreporting of self-reported exposures. The EuroJEM was developed by harmonizing JEM only from three Nordic countries. Due to differences in exposure definitions, metrics and occupational classifications systems, it was not possible to include JEM from other parts of Europe. Therefore, the EuroJEM may not be directly applicable in non-Nordic countries. Further research is needed to validate the EuroJEM in other regions and for different health-related outcomes. Additionally, EuroJEM includes only two exposures – high quantitative job demands and low decision authority – and could be expanded by including, for example, emotional job demands. The results might differ for quantitative and emotional job demands. Additionally, the healthy worker effect may have influenced the observed associations. Our study included only depression diagnosed in specialized healthcare, thus excluding diagnoses from other healthcare services. Further, since individuals with low socioeconomic status are more likely to suffer from undiagnosed depression, they are likely underrepresented in the outcome of the study. Lastly, there are some country differences in the adaptation and use of ICD-10 codes, which can affect the comparability between countries regarding the diagnostic depression rates (43).

Despite these limitations, this study has notable strengths. The construction of a cross-national and gender-specific JEM, harmonized by experts and applied in three large register-based cohorts with comparable results, is unique. The study used standardized design across cohorts, comparable register-based outcome, and consistent analytical approaches across cohorts to test the associations between exposures and diagnosed depression.

In conclusion, the EuroJEM performed similarly across national cohorts at least for decision authority and job strain, despite differences in the study populations, exposure distribution as well as prevalence of outcome. It is a valuable tool for assessing psychosocial job exposures in large-scale epidemiological studies, especially when individual-level data or a national JEM for the psychosocial work environment is unavailable. Sensitivity analyses suggested that adjustment for education when examining associations between JEM-based psychosocial exposures and depression might increase comparability of results across different studies. However, its performance is strongest (ie, consistently confirming previous findings) for decision authority and job strain, while caution is advised when using it to measure exposure to job demands.

## Supplementary material

Supplementary material

## Availability of EuroJEM

The EuroJEM is available for researchers at no cost after an application process. Information can be found at https://ki.se/en/imm/eurojem .

## References

[1] Boot CR, LaMontagne AD, Madsen IE. Fifty years of research on psychosocial working conditions and health: from promise to practice. Scand J Work Environ Health 2024 Sep;50(6):395–405. 10.5271/sjweh.4180 39110008 PMC11389251

[2] Niedhammer I, Bertrais S, Witt K. Psychosocial work exposures and health outcomes: a meta-review of 72 literature reviews with meta-analysis. Scand J Work Environ Health 2021 Oct;47(7):489–508. 10.5271/sjweh.3968 34042163 PMC8504166

[3] Rugulies R, Aust B, Greiner BA, Arensman E, Kawakami N, LaMontagne AD et al. Work-related causes of mental health conditions and interventions for their improvement in workplaces. Lancet 2023 Oct;402(10410):1368–81. 10.1016/S0140-6736(23)00869-3 37838442

[4] Mikkelsen S, Coggon D, Andersen JH, Casey P, Flachs EM, Kolstad HA et al. Are depressive disorders caused by psychosocial stressors at work? A systematic review with metaanalysis. Eur J Epidemiol 2021 May;36(5):479–96. 10.1007/s10654-021-00725-9 33580479 PMC8159794

[5] Podsakoff PM, MacKenzie SB, Podsakoff NP. Sources of method bias in social science research and recommendations on how to control it. Annu Rev Psychol 2012;63(1):539–69. 10.1146/annurev-psych-120710-100452 21838546

[6] Theorell T, Hasselhorn HM. On cross-sectional questionnaire studies of relationships between psychosocial conditions at work and health--are they reliable? Int Arch Occup Environ Health 2005 Aug;78(7):517–22. 10.1007/s00420-005-0618-6 15995878

[7] Fadel M, Evanoff BA, Andersen JH, d’Errico A, Dale AM, Leclerc A et al. Not just a research method: if used with caution, can job-exposure matrices be a useful tool in the practice of occupational medicine and public health? Scand J Work Environ Health 2020 Sep;46(5):552–3. 10.5271/sjweh.3900 32367143 PMC7737790

[8] Solovieva S, Roquelaure Y. Psychosocial Work Environment and Health: Applying Job-Exposure Matrices and Work Organization and Management Practice. In: Wahrendorf M, Chandola T, Descatha A, editors. Handbook of Life Course Occupational Health. Cham: Springer International Publishing; 2023.

[9] Descatha A, Evanoff BA, Leclerc A. Job-Exposure Matrices: Design, Validation, and Limitations. In: Wahrendorf M, Chandola T, Descatha A, editors. Handbook of Life Course Occupational Health. Cham: Springer International Publishing; 2023. p. 1–18. (Handbook Series in Occupational Health Sciences).

[10] Tielemans E, Heederik D, Burdorf A, Vermeulen R, Veulemans H, Kromhout H et al. Assessment of occupational exposures in a general population: comparison of different methods. Occup Environ Med 1999 Mar;56(3):145–51. 10.1136/oem.56.3.145 10448321 PMC1757718

[11] Wieclaw J, Agerbo E, Mortensen PB, Burr H, Tuchsen F, Bonde JP. Psychosocial working conditions and the risk of depression and anxiety disorders in the Danish workforce. BMC Public Health 2008 Aug;8(1):280. 10.1186/1471-2458-8-280 18687116 PMC2519085

[12] Rijs KJ, van der Pas S, Geuskens GA, Cozijnsen R, Koppes LL, van der Beek AJ et al. Development and validation of a physical and psychosocial job-exposure matrix in older and retired workers. Ann Occup Hyg 2014 Mar;58(2):152–70. 10.1093/annhyg/met052 24190953

[13] Karasek RA. Job Demands, Job Decision Latitude, Mental Strain: implications for job redesign. Adm Sci Q 1979;24(2):285. 10.2307/2392498

[14] Madsen IE, Gupta N, Budtz-Jørgensen E, Bonde JP, Framke E, Flachs EM et al. Physical work demands and psychosocial working conditions as predictors of musculoskeletal pain: a cohort study comparing self-reported and job exposure matrix measurements. Occup Environ Med 2018 Oct;75(10):752–8. 10.1136/oemed-2018-105151 30045952 PMC6166595

[15] Almroth M, Hemmingsson T, Sörberg Wallin A, Kjellberg K, Burström B, Falkstedt D. Psychosocial working conditions and the risk of diagnosed depression: a Swedish register-based study. Psychol Med 2021 Mar;52(15):1–9. 33682646 10.1017/S003329172100060X PMC9772906

[16] Kauppinen T, Mattila-Holappa P, Perkiö-Mäkelä M, Saalo A, Toikkanen J, Tuomivaara S et al. Työ ja terveys Suomessa 2012. Seurantatietoa työoloista ja työhyvinvoinnista. *[Work and health in Finland 2012. Follow-up data on working conditions and occupational wellbeing.]* Finnish Institute of Occupational Health, Helsinki: Tammerprint Oy; 2013.

[17] Solovieva S, Pensola T, Kausto J, Shiri R, Heliövaara M, Burdorf A et al. Evaluation of the Validity of Job Exposure Matrix for Psychosocial Factors at Work. Coyne J, editor. PLoS ONE. 2014;9(9):e108987. 10.1371/journal.pone.0108987 PMC4182611 25268276

[18] García AM, González-Galarzo MC, Kauppinen T, Delclos GL, Benavides FG. A job-exposure matrix for research and surveillance of occupational health and safety in Spanish workers: MatEmESp. Am J Ind Med 2013 Oct;56(10):1226–38. 10.1002/ajim.22213 23818037

[19] Hanvold TN, Sterud T, Kristensen P, Mehlum IS. Mechanical and psychosocial work exposures: the construction and evaluation of a gender-specific job exposure matrix (JEM). Scand J Work Environ Health 2019 May;45(3):239–47. 10.5271/sjweh.3774 30614504

[20] Niedhammer I, Chastang JF, Levy D, David S, Degioanni S, Theorell T. Study of the validity of a job-exposure matrix for psychosocial work factors: results from the national French SUMER survey. Int Arch Occup Environ Health 2008 Oct;82(1):87–97. 10.1007/s00420-008-0311-7 18327603

[21] Niedhammer I, Milner A, LaMontagne AD, Chastang JF. Study of the validity of a job-exposure matrix for the job strain model factors: an update and a study of changes over time. Int Arch Occup Environ Health 2018 Jul;91(5):523–36. 10.1007/s00420-018-1299-2 29520473

[22] Cohidon C, Santin G, Chastang JF, Imbernon E, Niedhammer I. Psychosocial exposures at work and mental health: potential utility of a job-exposure matrix. J Occup Environ Med 2012 Feb;54(2):184–91. 10.1097/JOM.0b013e31823fdf3b 22249578

[23] Peters S. Although a valuable method in occupational epidemiology, job-exposure -matrices are no magic fix. Scand J Work Environ Health 2020 May;46(3):231–4. 10.5271/sjweh.3894 32356897

[24] Pronk A, Loh M, Kuijpers E, Albin M, Selander J, Godderis L et al.; EPHOR Consortium. Applying the exposome concept to working life health: the EU EPHOR project. Environ Epidemiol 2022 Feb;6(2):e185. 10.1097/EE9.0000000000000185 35434456 PMC9005258

[25] Solovieva S, Descatha A, Mehlum IS, Viikari-Juntura E, Undem K, Berglund K et al. Development of a gender-specific European job exposure matrix (EuroJEM) for physical workload and its validation against musculoskeletal pain. Scand J Work Environ Health 2025 Mar;51(2):119–29. 10.5271/sjweh.4203 39680844 PMC11895771

[26] Solovieva S, Undem K, Falkstedt D, Johansson G, Kristensen P, Pedersen J et al. Utilizing a Nordic Crosswalk for Occupational Coding in an Analysis on Occupation-Specific Prolonged Sickness Absence among 7 Million Employees in Denmark, Finland, Norway and Sweden. Int J Environ Res Public Health 2022 Nov;19(23):15674. 10.3390/ijerph192315674 36497749 PMC9737405

[27] Ervasti J, Kausto J, Koskinen A, Pentti J, Vahtera J, Joensuu M et al. Labor Market Participation Before and After Long-Term Part-Time Sickness Absence in Finland: A Population-Based Cohort Study. J Occup Environ Med 2020 Apr;62(4):e142–8. 10.1097/JOM.0000000000001818 32032184

[28] Hasting RL, Merkus SL, Undem K, Kirkeleit J, Hoff R, Gran JM et al. Cohort Profile: The Nor-Work Cohort. Int J Epidemiol 2025 Feb;54(2):dyaf019. 10.1093/ije/dyaf019 40037803 PMC11879514

[29] Falkstedt D, Hemmingsson T, Albin M, Bodin T, Ahlbom A, Selander J et al. Disability pensions related to heavy physical workload: a cohort study of middle-aged and older workers in Sweden. Int Arch Occup Environ Health 2021 Nov;94(8):1851–61. 10.1007/s00420-021-01697-9 33880628 PMC8490214

[30] Higgins JP, Thompson SG, Spiegelhalter DJ. A re-evaluation of random-effects meta-analysis. J R Stat Soc Ser A Stat Soc 2009 Jan;172(1):137–59. 10.1111/j.1467-985X.2008.00552.x 19381330 PMC2667312

[31] Falkstedt D, Almroth M, Hemmingsson T, d’Errico A, Albin M, Bodin T et al. Job demands and job control and their associations with disability pension-a register-based cohort study of middle-aged and older Swedish workers. Int Arch Occup Environ Health 2023 Oct;96(8):1137–47. 10.1007/s00420-023-01995-4 37450035 PMC10504155

[32] Le GH, Hermansen Å, Dahl E. Constructing and validating an occupational job strain index based on five Norwegian nationwide surveys of living conditions on work environment. BMC Public Health 2023 Jan;23(1):50. 10.1186/s12889-022-14957-1 36609263 PMC9825027

[33] Hodson R. Gender Differences in Job Satisfaction: Why Aren’t Women More Dissatisfied? Sociol Q 1989;30(3):385–99. 10.1111/j.1533-8525.1989.tb01527.x

[34] Svane-Petersen AC, Holm A, Burr H, Framke E, Melchior M, Rod NH et al. Psychosocial working conditions and depressive disorder: disentangling effects of job control from socioeconomic status using a life-course approach. Soc Psychiatry Psychiatr Epidemiol 2020 Feb;55(2):217–28. 10.1007/s00127-019-01769-9 31506742

[35] Theorell T, Hammarström A, Aronsson G, Träskman Bendz L, Grape T, Hogstedt C et al. A systematic review including meta-analysis of work environment and depressive symptoms. BMC Public Health 2015 Aug;15(1):738. 10.1186/s12889-015-1954-4 26232123 PMC4522058

[36] Madsen IE, Nyberg ST, Magnusson Hanson LL, Ferrie JE, Ahola K, Alfredsson L et al.; IPD-Work Consortium. Job strain as a risk factor for clinical depression: systematic review and meta-analysis with additional individual participant data. Psychol Med 2017 Jun;47(8):1342–56. 10.1017/S003329171600355X 28122650 PMC5471831

[37] Seidler A, Schubert M, Freiberg A, Drössler S, Hussenoeder FS, Conrad I et al. Psychosocial occupational exposures and mental illness—a systematic review with meta-analyses. Dtsch Arztebl Int 2022 Oct;119(42):709–15. 10.3238/arztebl.m2022.0295 36345690 PMC9835701

[38] Mäntyniemi A, Oksanen T, Salo P, Virtanen M, Sjösten N, Pentti J et al. Job strain and the risk of disability pension due to musculoskeletal disorders, depression or coronary heart disease: a prospective cohort study of 69,842 employees. Occup Environ Med 2012 Aug;69(8):574–81. 10.1136/oemed-2011-100411 22573793

[39] Podsakoff NP, LePine JA, LePine MA. Differential challenge stressor-hindrance stressor relationships with job attitudes, turnover intentions, turnover, and withdrawal behavior: a meta-analysis. J Appl Psychol 2007 Mar;92(2):438–54. 10.1037/0021-9010.92.2.438 17371090

[40] Grynderup MB, Mors O, Hansen ÅM, Andersen JH, Bonde JP, Kærgaard A et al. A two-year follow-up study of risk of depression according to work-unit measures of psychological demands and decision latitude. Scand J Work Environ Health 2012 Nov;38(6):527–36. 10.5271/sjweh.3316 22885721

[41] Bonde JP. Psychosocial factors at work and risk of depression: a systematic review of the epidemiological evidence. Occup Environ Med 2008 Jul;65(7):438–45. 10.1136/oem.2007.038430 18417557

[42] Melchior M, Caspi A, Milne BJ, Danese A, Poulton R, Moffitt TE. Work stress precipitates depression and anxiety in young, working women and men. Psychol Med 2007 Aug;37(8):1119–29. 10.1017/S0033291707000414 17407618 PMC2062493

[43] Quan H, Moskal L, Forster AJ, Brien S, Walker R, Romano PS et al. International variation in the definition of ‘main condition’ in ICD-coded health data. Int J Qual Health Care 2014 Oct;26(5):511–5. 10.1093/intqhc/mzu064 24990594 PMC4207866

[44] Statistics Sweden SC. SSYK 1996 Standard for svensk yrkesklassificering 1996 (Swedish Standard Classification of Occupations 1996). 2001. https://www.scb.se/contentassets/50bf86d2c66149d696f515a9481bc9e2/ov9999_1998a01_br_x70op9803.pdf

