# Supplementary material for: Development of a European job exposure matrix (EuroJEM) for psychosocial exposures and their association with diagnosed depression in register-based cohorts
Source: Scand J Work Environ Health. 2026 Apr 30;52(3):252–62. doi: 10.5271/sjweh.4279 (PMC13182255; doi:10.5271/sjweh.4279)
Supplement: Supplementary material [file SJWEH-52-252-S001.pdf]

Development of a European job exposure matrix (EuroJEM) for psychosocial exposures and their association with diagnosed depression in register-based cohorts[1]

by Laura Salonen, PhD,*[2] *Daniel Falkstedt, PhD, Kuan-Yu Pan, PhD, Maria Albin, PhD, Ingrid Sivesind Mehlum, PhD, Karina Undem, MPhil, Taina Leinonen, PhD, Svetlana Solovieva, PhD

1. SUPPLEMENTARY MATERIAL
2. Corresponding author: Laura Salonen, PhD, Finnish Institute of Occupational Health, PL 40, 00032 Työterveyslaitos Finland. [E-mail: laura.salonen@ttl.fi]

Supplementary Table S1. Overview of identified JEMs for psychosocial exposures.

JEM name, country	Exposures	Exposure categorization	Occupational codes
Psychosocial JEM, Denmark[1]<br>Madsen et al. 2018	Quantitative demands: Work time, work pace, unclear tasks (5 items)<br>Emotional demands: Emotional involvement at work (2 items)<br>Decision authority influence on task performance (2 items)<br>Job strain: Strain variable was dichotomized; jobs with the prevalence of strain higher than 20% were defined as high strain job<br>Job insecurity: Worry about unemployment<br>Work-related violence: being exposed to violence within 12 months	Exposure scales ranged between 1–5 for each exposure, except work-related violence and job insecurity (that were dichotomised). For each item a mean score was calculated.	DISCO-08, comparable with ISCO-08
Psychosocial working conditions JEM, Denmark[2]<br>Wieclaw et al. 2008	Emotional demands: emotional situations, emotional involvement (3 items)<br>Working with people: work with people (1 item)<br>Quantitative demands, decision authority	Data regarding the demand-control model were collected on the basis of an adopted version of the Job Content Questionnaire [1] containing five 3–5 questions scales and single item questions with 4–6 response options. Based on individual scores, the mean was calculated for each occupational group.	DISCO-88, comparable with ISCO-88

JEM for psychosocial factors, Finland[3] Solovieva et al. 2014	Job demands: work pace, work load (5 items)<br>job control: decision authority: free and a lot to say on the job (3 items); skills discretion: learning, creativity, development (5 items)<br>monotonous work<br>social support: supervisors, co-workers, discussion (4 items)<br>job strain	Each item was measured in the scale of 1-5. Gender-specific median cut-off points were used to dichotomize job demands, job control and monotonous work.	National occupational classification, comparable with ISCO-88
FINJEM, Finland[4] Kauppinen et al. 1998	Challenge at work; Social climate at work; Control possibilities at work; Work load; Risks at work; Social demands at work; Supervisor support at work; Working time arrangements	The aggregate variables were scaled back to the original scale 1–5 used in the survey. All workers were considered “exposed’’ to most psychosocial factors, but the level varied between one and five. The final estimates were rounded to the closest integer.	Own classification, based on ISCO-58
SUMMER JEM-2008, France[5,6] Niedhammer et al. 2008; 2018	Psychological work demands: Skill discretion (6 items) and decision authority (3 items)<br>Job strain<br>Social support (8 items)	Each item was summed up for psychological demands and social support, and weights were introduced for the score of decision authority to give the same weight to the two sub-dimensions of skill discretion and decision authority. The individual scores were thereafter dichotomized at the median of the total sample.	French 4-digit Profession et Catégorie Sociale (PCS) 2003 job codes and economic activities (NAF) 2003
Psychosocial JEM, Norway[7] Hanvold et al. 2019	Quantitative demands: skip break or work longer (1 item)<br>Role conflict: contradictory requests, insufficient tools, organizing work (3 items)<br>Emotional demand: Deal with emotions (1 item)<br>Decision authority: work pace, organizing work (3 items)	Each item was measured in the scale of 1-5. Gender-specific median cut-off points were used to dichotomize job demands, job control and monotonous work. the JEM exposure scores for psychosocial work exposures were calculated as the mean of the	STYRK-98, comparable with ISCO-88

	Skill discretion: opportunities for skill development (2 items)<br>Supportive leadership: support, appreciation and fairness (3 items)<br>Monotonous work. task repetition (1 item)<br>Job strain	dichotomized individual score in each occupational group .	
MatEmEsp, Spain[8]<br>García et al. 2013	Based on FINJEM (Solovieva et al. 2014): work pace, quantitative demands, emotional demands, influence at work, skill development, supervisor support, coworker support, job insecurity, esteem	Standardized scores (mean, median, standard deviation minimum, and maximum values) on a scale of 0–100 were used as indicators of exposure.	CNO-94, a national system based on ISCO-88
Work organization matrix (WOM), Sweden[9]<br>Johnson et al. 1990	Psychological work demands (2 items), job control (12 items on decision authority and skill discretion) and social support (4 items)	Exposure scores for each job characteristic were estimated by gender, occupation, age categories, and the duration of time in the specific occupation.	National classification system
Psychosocial JEM 2000, Sweden[10]<br>Fredlund et al. 2000	Psychological work demands, job control,	A mean score (range 0–10), based on occupation, sex, and age for job demands and control and social support.	SSYK -96, comparable with ISCO-88
Psychosocial SWEJEM, Sweden[11]<br>Almroth et al. 2021	Psychological work demands, job control, social support<br>Decision authority: work pace, work time, organizing work (4 items)<br>Quantitative demands: stress, skip break or work longer, require concentration (3 items)<br>Job strain	The measures were scored as a mean for each occupation. Then the mean scores were categorized according to their quintile distribution separately for men and women, resulting in five categories ranging from low to high.	SSYK -96, comparable with ISCO-88
GPJEM, The Netherlands[12]<br>Rijs et al. 2014	Job demands: Time pressure, task requirements, cognitive demands<br>Psychological resources: Autonomy, variation activities<br>Social support: Co-worker support, supervisor support	Sum scores were calculated of all items for each exposure. Sum scores were dichotomized based on the median value.	NSCO-92, can be linked to ISCO-08
**References**			

1. Madsen IEH, Gupta N, Budtz-Jørgensen E, Bonde JP, Framke E, et al. Physical work demands and psychosocial working conditions as predictors of musculoskeletal pain: a cohort study comparing self-reported and job exposure matrix measurements. Occup Environ Med 2018; 75(10):752-8.
2. Wieclaw J, Agerbo E, Mortensen PB, Burr H, Tuchsen F, Bonde JP. Psychosocial working conditions and the risk of depression and anxiety disorders in the Danish workforce. BMC Public Health 2008; 8:280.
3. Solovieva S, Pensola T, Kausto J, Shiri R, Heliövaara M, et al. Evaluation of the validity of job exposure matrix for psychosocial factors at work. PLoS One 2014; 9(9):e108987.
4. Kauppinen T, Toikkanen J, Pukkala E. Am From cross-tabulations to multipurpose exposure information systems: a new job-exposure matrix. J Ind Med 1998; 33(4):409-17.
5. Niedhammer I, Chastang JF, Levy D, David S, Degioanni S, Theorell T. Study of the validity of a job-exposure matrix for psychosocial work factors: results from the national French SUMER survey. Int Arch Occup Environ Health 2008; 82(1):87-97.
6. Niedhammer I, Milner A, LaMontagne AD, Chastang JF. Study of the validity of a job-exposure matrix for the job strain model factors: an update and a study of changes over time. Int Arch Occup Environ Health 2018; 91(5):523-536.
7. Hanvold TN, Sterud T, Kristensen P, Mehlum IS. Mechanical and psychosocial work exposures: the construction and evaluation of a gender-specific job exposure matrix (JEM). Scand J Work Environ Health 2019; 45(3):239-247.
8. García AM, González-Galarzo MC, Kauppinen T, Delclos GL, Benavides FG. A job-exposure matrix for research and surveillance of occupational health and safety in Spanish workers: MatEmESp. Am J Ind Med 2013; 56(10):1226-38.
9. Johnson JV, Stewart W, Fredlund P, Hall EM, Theorell T. Psychosocial job exposure matrix: an occupationally aggregated attribution system for work environment exposure characteristics. National Institute for psychosocial factors and health. WHO Psychosocial Centre: Department of Stress Research; 1990.
10. Fredlund P, Hallqvist J, Diderichsen F. Psychosocial job exposure matrix. An updated version of a classification system for work-related psychosocial exposure. Stockholm, Sweden: Swedish National Institute for Working Life, National Institute for Working Life; 2000.
11. Almroth M, Hemmingsson T, Sörberg Wallin A, Kjellberg K, et al. Psychosocial working conditions and the risk of diagnosed depression: a Swedish register-based study. Psychol Med 2021 8:1-9.
12. Rijs KJ, van der Pas S, Geuskens GA, Cozijnsen R, Koppes LL, et al. Development and Validation of a Physical and Psychosocial Job-Exposure Matrix in Older and Retired Workers. Ann Occup Hyg 2014; 58(2):152-70.

Supplementary Table S2. Definitions of exposures selected for harmonization and the harmonization procedure.

Exposure	National JEMs			EuroJEM
	Finnish	Norwegian	Swedish	
**Quantitative job demands**	Not enough time;<br>No excessive work;<br>Work fast	Not able to finish all work and necessary to skip lunch/work late	Do you sometimes have so much to do that you have to pull in for lunches, work over or take work home?<br>So stressful not having time to talk or think about anything other than work	Time pressure
	Work hard / intense concentration		Does the work require all your attention and concentration	Mental workload
	Conflicting demands / hectic work			Conflicting demands/hectic work
**Decision authority (job control)**	Allows own decisions	Allows own decisions	Decide work pace, decide when to take breaks, decide when to do tasks	Allows own decisions
	Little decision freedom	Little decision freedom	Little decision freedom	Little decision freedom
	A lot to say		Influence planning of own work	A lot to say

Supplementary Table S3. Number (%) of ISCO-88 (COM) occupational groups by exposure category in the national JEMs

	Men			Women		
	Finnish JEM (N=332)*	Norwegian JEM (N=341)	Swedish JEM (N=365)	Finnish JEM (N=328)	Norwegian JEM (N=320)	Swedish JEM (N=365)
Exposure/categories	N (%)	N (%)	N (%)	N (%)	N (%)	N (%)
High job demand						
0-24%	3 (0.9)	52 (15.3)	80 (21.9)	12 (3.7)	55 (17.2)	114 (31.2)
25-49%	153 (46.1)	162 (47.5)	240 (65.8)	140 (42.7)	147 (45.9)	195 (53.4)
50-74%	152 (45.8)	111 (32.6)	44 (12.1)	142 (43.3)	105 (32.8)	56 (15.3)
75-100%	24 (7.2)	16 (4.7)	1 (0.3)	34 (10.4)	13 (4.1)	0 (0.0)
Low decision authority						
0-24%	53 (16.0)	28 (8.2)	74 (20.3)	49 (14.9)	22 (6.9)	147 (40.3)
25-49%	80 (24.1)	161 (47.2)	198 (54.3)	64 (19.5)	136 (42.5)	160 (43.8)
50-74%	149 (44.9)	142 (41.6)	90 (24.7)	143 (43.6)	149 (46.6)	54 (14.8)
75-100%	50 (15.1)	10 (2.9)	3 (0.8)	72 (22.0)	13 (4.1)	4 (1.1)

*Total number of ISCO-88 (COM) occupational groups with exposure estimates in the national JEM.

Supplementary Table S4. Number (%) of occupational codes with agreement/disagreement in exposure categories between the national JEMs among men.

Exposure	Full agreement	Some agreement	Disagreement	Codes missing*	Examples**
	N (%)	N (%)	N (%)	N (%)	Codes
**Men**					
Job demands	55 (14.8)	247 (66.6)	54 (14.6)	15 (4.0)	1228, 2211, 3411, 5113
Decision authority	68 (18.3)	216 (58.2)	72 (19.4)	15 (4.0)	2111, 2131, 2332, 3443
**Women**					
Job demands	49 (13.2)	221 (59.6)	86 (23.2)	15 (3.8)	1231, 2223, 3422, 8274
Decision authority	29 (7.8)	186 (50.1)	141 (38.0)	15 (4.0)	2351, 3222, 3433, 4223

*Number of occupational ISCO-88 (COM) which cannot be matched to the national codes.
** Examples of code with disagreement.
Full agreement- the same exposure category in all three national JEMs; some agreement – the same exposure category in at least two national JEMs; disagreement – different exposure categories in the national JEMs.

**ISCO88(COM) code**	**Title**
1228	Production and operations managers in personal care, cleaning and related services
1231	Finance and administration managers
2111	Physicists and astronomers
2131	Computer systems designers, analysts and programmers
2211	Biologists, botanists, zoologists and related professionals
2223	Veterinarians

2332	Pre-primary education teaching professionals
2351	Education methods specialists
3222	Hygienists, health and environmental officers
3411	Securities and finance dealers and brokers
3422	Clearing and forwarding agents
3433	Bookkeepers
3443	Government social benefits officials
4223	Telephone switchboard operators
5113	Travel guides
8274	Baked-goods, cereal- and chocolate-products machine operators

Supplementary Table S5. Concordance of exposure assessment between the national JEMs and the EuroJEM. Number of occupational codes with the same exposure category in the national JEM and EuroJEM.

	Men			Women		
	Finnish JEM	Norwegian JEM	Swedish JEM	Finnish JEM	Norwegian JEM	Swedish JEM
Exposure/categories	N (%)	N (%)	N (%)	N (%)	N (%)	N (%)
High job demand						
0-24%	1 (0.3)	34 (9.9)	40 (10.8)	7 (2.1)	32 (9.9)	41 (11.1)
25-49%	127 (37.3)	149 (43.3)	196 (53.1)	111 (33.5)	135 (41.7)	158 (42.8)
50-74%	64 (19.1)	71 (20.6%)	36 (9.8)	59 (17.8)	77 (23.8)	51 (13.8)
75-100%	2 (0.6)	0 (0.0%)	0 (0.0)	3 (0.9)	3 (0.9)	0 (0.0)
Accuracy[1]	194 (57.3)	254 (73.8)	272 (73.7)	180 (54.3)	247 (76.3)	250 (69.5)
Kappa	0.28	0.59	0.54	0.26	0.61	0.46
Low decision authority						
0-24%	29 (8.7)	25 (7.3)	38 (10.3)	37 (11.2)	22 (6.8)	48 (13.0)
25-49%	68 (20.3)	138 (40.1)	124 (33.6)	57 (17.2)	108 (33.4)	81 22.0)
50-74%	84 (25.1)	119 (34.6)	77 (20.9)	70 (21.1)	106 (32.8)	47 (12.7)
75-100%	6 (1.8)	3 (0.9)	3 (0.8)	1 (0.3)	4 (1.9)	3 (0.8)
Accuracy[1]	187 (55.9)	285 (75.9)	242 (65.6)	165 (40.7)	240 (74.9)	179 (48.5)
Kappa[2]	0.35	0.72	0.45	0.32	0.58	0.25

[1] Classification accuracy shows proportion of occupations categorized similarly in the national and developed JEM

[2] Cohen's Kappa coefficients: poor (<0.20), fair (0.20-0.40), moderate (0.41-0.60), good (0.61-0.80) and excellent (0.81-1.0) agreement.

Cohen's kappa values varied from good to fair. Regarding high job demands categories, the agreement was good in Norwegian JEM (women), moderate in Swedish JEM (both genders), and in Norwegian JEM (men), and fair in Finnish JEM (both genders). Low decision authority agreement was good in Norwegian men, moderate in Norwegian women and Swedish men, and fair for Finnish men and women and for Swedish women.

Supplementary Table S6. ISCO-88 (COM) occupations in the highest category of exposures (75-100%)

Code	Title	High job demands	Low decision authorities
1110	Legislators and senior government officials	men	
1141	Senior officials of political party organizations	men	
1142	Senior officials of employers', workers' and other economic-interest organizations	men	
1143	Senior officials of humanitarian and other special-interest organizations	men	
1223	Production and operations managers in construction	both genders	
1236	Computing services managers	women	
2221	Medical doctors	women	
4223	Telephone switchboard operators		women
5111	Travel attendants and travel stewards		men
5123	Waiters, waitresses and bartenders		men
7413	Dairy-products workers		both genders
7414	Fruit, vegetable and related preservers		women
8272	Dairy-products machine operators		both genders
8275	Fruit-, vegetable- and nut-processing-machine operators		women
8311	Locomotive engine drivers		both genders
8323	Bus and tram drivers		men
9113	Door-to-door and telephone salespersons		women

Supplementary Table S7. Table 1 with four categories.

Table 1. Version with four categories.							
		Men			Women		
Variable	Categories	Finnish	Norwegian	Swedish	Finnish	Norwegian	Swedish
		%	%	%	%	%	%
**High job demands**							
Low likelihood	0-24%	4.4	5.3	7.1	3.7	3.3	7.9
Medium likelihood	25-49%	68.6	68.5	70.2	65.0	66.7	69.1
Medium high likelihood	50-74%	26.5	25.7	22.5	29.9	29.3	22.2
High likelihood	75-100%	0.5	0.5	0.2	1.3	0.8	0.8
**Low decision authority**							
Low likelihood	0-24%	8.1	12.6	9.1	5.8	7.2	9.9
Medium likelihood	25-49%	54.8	54.5	54.0	46.7	46.8	53.2
Medium high likelihood	50-74%	36.1	31.1	35.3	46.7	45.2	36.0
High likelihood	75-100%	1.0	1.8	1.6	0.9	0.8	0.9

Supplementary Table S8. Hazard ratios (HR) and 95% confidence intervals (CI) of the association between EuroJEM-based psychosocial exposures and the risk of diagnosed depression among men using four exposure categories.

								Meta-analysis (Model 2)			
**Exposure**	**Category**	**Model 1: Age-adjusted**			**Model 2: Age- and education-adjusted**			**Meta-analysis (Model 2)**			
		**Finnish**	**Norwegian**	**Swedish**	**Finnish**	**Norwegian**	**Swedish**				
		**HR (95% CI)**	**HR (95% CI)**	**HR (95% CI)**	**HR (95% CI)**	**HR (95% CI)**	**HR (95% CI)**	**HR (95% CI)**	**I^2**	**Q (p-value)**	**τ**
**High job demands**											
Low likelihood	0-24%	1.00	1.00	1.00	1.00	1.00	1.00	1.00			
Medium likelihood	25-49%	1.16 (1.09-1.23)	1.01 (0.96-1.05)	1.01 (0.98-1.04)	1.22 (1.15-1.30)	1.05 (1.01-1.10)	1.07 (1.04-1.11)	1.11 (1.03-1.19)	82.4%	17.10 (0.0003)	0.06
Medium-high likelihood	50-74%	0.81 (0.75-0.86)	0.82 (0.79-0.86)	0.69 (0.67-0.72)	0.97 (0.90-1.04)	0.91 (0.87-0.95)	0.81 (0.78-0.84)	0.89 (0.80-0.99)	88.8%	26.8 (< 0.0001)	0.09
High likelihood	75-100%	0.56 (0.43-0.72)	0.65 (0.54-0.78)	0.40 (0.31-0.52)	0.70 (0.55-0.91)	0.72 (0.60-0.86)	0.48 (0.37-0.62)	0.63 (0.49-0.80)	53.3%	6.87 (0.032)	0.18
**Low decision authority**											
Low likelihood	0-24%	1.00	1.00	1.00	1.00	1.00	1.00	1.00			

Medium likelihood	25-49%	1.29 (1.22-1.36)	1.22 (1.17-1.26)	1.51 (1.46-1.57)	1.13 (1.07-1.20)	1.17 (1.14-1.22)	1.38 (1.33-1.44)	1.22 (1.08-1.38)	93.9%	49.0 (< 0.0001)	0.10
Medium-high likelihood	50-74%	1.67 (1.58-1.77)	1.56 (1.50-1.61)	1.90 (1.83-1.97)	1.35 (1.28-1.44)	1.45 (1.40-1.51)	1.67 (1.61-1.74)	1.19 (1.32-1.67)	93.2%	44.1 (< 0.0001)	0.10
High likelihood	75-100%	2.35 (2.11-2.63)	1.86 (1.74-1.99)	2.21 (2.07-2.36)	1.86 (1.67-2.08)	1.69 (1.58-1.81)	1.92 (1.80-2.05)	1.82 (1.67-1.98)	58.9%	7.30 (0.026)	0.06

Supplementary Table S9. Hazard ratios and 95% confidence intervals of the association between EuroJEM-based psychosocial exposures and the risk of diagnosed depression among women using four exposure categories.

**Exposure**	**Category**	**Model 1: Age adjusted**			**Model 2: Age and education adjusted**			**Meta-analysis (Model 2)**			
		**Finnish**	**Norwegian**	**Swedish**	**Finnish**	**Norwegian**	**Swedish**				
		**HR (95% CI)**	**HR (95% CI)**	**HR (95% CI)**	**HR (95% CI)**	**HR (95% CI)**	**HR (95% CI)**	**HR (95% CI)**	**I^2**	**Q (p-value)**	**τ**
**High job demands**											
Low likelihood	0-24%	1.00	1.00	1.00	1.00	1.00	1.00	1.00			
Medium likelihood	25-49%	1.22 (1.16-1.29)	1.18 (1.13-1.25)	1.00 (0.98-1.02)	1.26 (1.20-1.33)	1.21 (1.15-1.27)	1.07 (1.04-1.09)	1.18 (1.05-1.31)	93.2 %	43.9 (< 0.0001)	0.09
Medium-high likelihood	50-74%	0.97 (0.92-1.03)	1.10 (1.04-1.15)	0.74 (0.72-0.76)	1.15 (1.09-1.22)	1.16 (1.10-1.22)	0.91 (0.89-0.94)	1.07 (0.89-1.28)	97.0 %	99.6 (< 0.0001)	0.16
High likelihood	75-100%	0.74 (0.66-0.83)	1.18 (1.07-1.30)	1.10 (1.03-1.17)	0.94 (0.84-1.06)	1.25 (1.13-1.38)	1.48 (1.38-1.59)	1.21 (0.94-1.55)	93.1 %	43.3 (< 0.0001)	0.22
**Low decision authority**											
Low likelihood	0-24%	1.00	1.00	1.00	1.00	1.00	1.00	1.00			
Medium likelihood	25-49%	1.31 (1.25-1.38)	1.21 (1.17-1.25)	1.28 (1.25-1.31)	1.22 (1.16-1.28)	1.18 (1.14-1.22)	1.24 (1.21-1.27)	1.21 (1.18-1.25)	44.9 %	5.4 (0.065)	0.02

Medium-high likelihood	50-74%	1.59 (1.52-1.67)	1.28 (1.24-1.33)	1.37 (1.34-1.40)	1.38 (1.31-1.45)	1.26 (1.21-1.30)	1.24 (1.21-1.27)	1.29 (1.22-1.36)	78.5 %	13.9 (0.0009)	0.04
High likelihood	75-100%	1.90 (1.72-2.09)	1.51 (1.29-1.64)	1.44 (1.35-1.53)	1.60 (1.45-1.77)	1.44 (1.33-1.56)	1.27 (1.19-1.36)	1.42 (1.25-1.62)	80.6 %	15.4 (0.0004)	0.11

Supplementary Table S10. Associations between EuroJEM-based exposures and diagnosed depression by national cohort in men. Hazard ratios (HR) and 95% confidence intervals (CI).

Exposure	Category	Model 1: Age-adjusted			Meta-analysis (Model 1)			
		Finnish	Norwegian	Swedish				
		HR (95% CI)	HR (95% CI)	HR (95% CI)	HR (95% CI)	I^2	Q (p-value)	τ
**High job demands**								
Low likelihood	0-24%	1.00	1.00	1.00	1.00			
Medium likelihood	25-49%	1.16 (1.09-1.23)	1.01 (0.96-1.05)	1.01 (0.98-1.04)	1.05 (0.98-1.13)	82.6%	17.3 (0.0002)	0.06
Medium-high / high likelihood	50-100%	0.80 (0.75-0.86)	0.82 (0.78-0.86)	0.69 (0.67-0.71)	0.77 (0.68-0.87)	93.1%	43.2 (< 0.0001)	0.11
**Low decision authority**								
Low likelihood	0-24%	1.00	1.00	1.0	1.00			
Medium likelihood	25-49%	1.29 (1.22-1.36)	1.22 (1.17-1.26)	1.51 (1.46-1.57)	1.33 (1.18-1.52)	95.7%	70.0 (< 0.0001)	0.11
Medium-high / high likelihood	50-100%	1.67 (1.60-1.79)	1.57 (1.52-1.63)	1.91 (1.84-1.98)	1.71 (1.50-1.95)	95.3%	63.4 (< 0.0001)	0.11
**Job strain**								
	Low strain	1.00	1.00	1.0	1.00			

	Passive	1.20 (1.17-1.24)	1.28 (1.25-1.30)	1.21 (1.19-1.23)	1.23 (1.18-1.28)	86.6%	22.3 (0.0001)	0.3
	Active	0.71 (0.70-0.74)	0.82 (0.80-0.84)	0.68 (0.66-0.70)	0.73 (0.65-0.82)	97.2%	107.9 (< 0.0001)	0.10
	High strain	1.12 (1.07-1.17)	1.20 (1.15-1.25)	1.17 (1.09-1.25)	1.16 (1.11-1.22)	39.1%	4.9 (0.09)	0.03

Supplementary Table S11. Associations between EuroJEM-based exposures and diagnosed depression by national cohort in women. Hazard ratios (HR) and 95% confidence intervals (CI).

**Exposure**	**Category**	**Model 1: Age adjusted**			**Meta-analysis (Model 1)**			
		**Finnish**	**Norwegian**	**Swedish**				
		**HR (95% CI)**	**HR (95% CI)**	**HR (95% CI)**	**HR (95% CI)**	**I^2**	**Q (p-value)**	**τ**
**High job demands**								
Low likelihood	0-24%	1.00	1.00	1.00	1.00			
Medium likelihood	25-49%	1.22 (1.16-1.29)	1.19 (1.13-1.25)	1.00 (0.98-1.02)	1.13 (0.98-1.31)	96.1%	76.7 (< 0.0001)	0.13
Medium-high / high likelihood	50-100%	0.96 (0.91-1.02)	1.10 (1.05-1.15)	0.75 (0.73-0.77)	0.92 (0.72-1.19)	98.7%	225.2 (< 0.0001)	0.23
**Low decision authority**								
Low likelihood	0-24%	1.00	1.00	1.00	1.00			
Medium likelihood	25-49%	1.31 (1.25-1.38)	1.21 (1.17-1.25)	1.28 (1.25-1.31)	1.26 (1.21-1.32)	69.4%	9.8 (0.007)	0.03
Medium-high / high likelihood	50-100%	1.60 (1.52-1.68)	1.29 (1.24-1.33)	1.37 (1.34-1.41)	1.41 (1.28-1.56)	93.8%	48.0 (< 0.0001)	0.09
**Job strain**								
	Low strain	1.00	1.00	1.00	1.00			

	Passive	1.17<br>(1.15-1.20)	1.07<br>(1.05-1.09)	1.04<br>(1.02-1.05)	1.09<br>(1.02-1.17)	96.3%	81.0<br>(< 0.0001)	0.06
	Active	0.81<br>(0.78-0.83)	0.90<br>(0.88-0.93)	0.73<br>(0.72-0.75)	0.81<br>(0.71-0.92)	97.9%	145.5<br>(< 0.0001)	0.11
	High strain	1.05<br>(1.01-1.08)	1.03<br>(1.01-1.06)	0.89<br>(0.86-0.92)	0.99<br>(0.90-1.09)	79.1%	59.7<br>(0.0008)	0.08

Supplementary Methods S1. Description of selected national JEMs.

The Finnish psychosocial JEM has been developed utilizing self-reported exposure information from the large and nationally representative Health 2000 Survey, conducted in 2000-2001 (17). The matrix includes five psychosocial exposures and covers 365 different occupational groups coded with the Finnish Standard Classification of Occupations 2001 coding system. The JEM showed good accuracy in identification of individuals exposed to high job strain, low decision authority and monotonous work, as well as a good predictive validity for self-reported depression and low back pain.

The Norwegian psychosocial JEM has been developed utilizing self-reported exposure information from the Norwegian nationwide Survey of Living Conditions on work environment, conducted in 2006 and 2009 (19). The matrix includes five psychosocial exposures and covers 268 different occupational groups coded with the Norwegian Standard Classification of Occupations (STYRK-98) coding system. The matrix showed a good predictive validity for low back pain.

The Swedish psychosocial (SWEJEM) has been constructed based on self-reported exposure information from the Swedish Work Environment Surveys measured during 1997-2013 (44). The matrix includes three exposures and covers 355 different occupational groups coded with the Swedish Standard Classification of Occupations (SSYK) 96 coding system. The matrix showed a good predictive validity for diagnosed depression (15).

Supplementary Methods S2. Harmonization procedure of the EuroJEM.

1. For each selected exposure in the national JEMs we categorized the proportion of exposed into four categories: 1=0-24%; 2=25-49%; 3=50-74%; 4=75-100%

2. The occupations were checked for agreement regarding exposure category between the JEMs.

   - When there **was full** agreement between JEMs for an occupation, this category value was assigned to the EuroJEM.

   - For occupations with **disagreement**s, the expert panel discussed until a consensus was eached, and then assigned exposure categories to the EuroJEM.

3. The following considerations were used in the consensus discussions:

   a. Tasks and activities within the occupation were discussed and used to resolve disagreements. Correspondence between the descriptions of the occupation under ISCO-88(COM) code and for matched national codes were also looked at. The ISCO-88 (COM) contains 372 4-digit occupational codes and the developed EuroJEM provides exposure categories for all 4-digit occupational codes, except "0100 Army force". This occupational group is relatively small compared to other occupational groups and very heterogeneous in composition. Discussions were held about whether the exposure categories of the different JEMs were reasonable according to what was known by the panel experts regarding work tasks and activities.

   b. Characteristics of the national JEMs:

      i. The size of the occupational group, i.e. the number of respondents from the particular occupation in the survey on which the exposure estimate in the national JEM was calculated. The following categories for the size of occupations were used: >199, 50- 199, 10-49 and < 10. JEMs where the estimate was based on smaller group sizes were considered less reliable. Estimates from JEMs with larger sizes had higher weights for the consensus agreements.

      ii. Attention was paid to whether there was a genuine estimate (based on survey responses for the specific occupation and gender) or whether the estimate was based on merged occupations or genders. JEMs with genuine estimates were considered more reliable.

   c. If disagreements were considered to reflect true differences between the countries regarding the work content in an occupation, and all values to be trustworthy with regard to i - ii. above, a mean value of the exposure categories was assigned. If one

national value was close to the border of the exposure category, and close to the other JEM values, this was also taken into consideration.

4. For occupations with missing information on exposure estimates from most of the national JEMs, the expert panel discussed until reaching a consensus and assigned exposure categories to these occupations. The same considerations described above (bullet 3) were used, with the addition of the following solutions when relevant:

    a. For occupations with agreement in exposure rates based on two JEMs and missing in the third JEM, the exposure category from the two JEMs was assigned.

    b. When exposure estimates were available from at least one national JEM, the exposure category from a JEM with a relatively large occupation size was assigned.

    c. If an exposure estimate was available for only one gender, it was considered to assign the same exposure category for both genders (a similar strategy was applied during the construction of the national JEMs)

Supplementary Methods S3. Description of the register-based cohorts.

The SLFF cohort (27) consists of a 90% nationally representative random sample of the Finnish working age population (18–68 years old) living in Finland on 31 December 2010. This sample was supplemented with a 90% sample of Finnish residents, who became of age 18 in each year between 2011 and 2019 (N ≈ 3.9 million). The data include information on diagnoses from in- and out- patient healthcare visits from the Care Register for Health Care and on sociodemographic factors and occupational titles obtained from the FOLK modules of Statistics Finland. Information on date of death was obtained from the Population Census register.

The Nor-Work cohort consists of all individuals born 1930–1992, who have resided or worked in Norway (N ≈ 5.8 million). The data include information on diagnoses from in- and out- patient healthcare visits from the Norwegian Patient Registry and on occupational titles, sociodemographic factors, and date of death obtained from Statistics Norway.

The SWIP cohort (29) consists of all individuals born 1941–1989 and residing in Sweden in 2005 and aged 16–64 that year (N ≈ 5.4 million). The data include information from several registers held by Statistics Sweden, such as sociodemographic factors, occupational titles and date of death from the Swedish register of the total population and the Longitudinal Integrated Database for Health Insurance and Labor Market Studies (LISA), and diagnoses from in- and out-patient healthcare visits from registers held by the National Board of Health and Welfare.
